# 中性粒细胞缺乏伴发热患者抗菌药物临床应用中国指南（2026年版）

**DOI:** 10.3760/cma.j.cn121090-20260120-00041

**Published:** 2026-04

**Authors:** 中华医学会血液学分会 中国医师协会血液科医师分会

## Abstract

中性粒细胞缺乏（粒缺）伴发热患者是一组特殊的疾病人群，如未及时给予恰当的抗菌药物治疗，感染相关死亡率高。近年来国际及国内关于粒缺伴发热的诊疗理念发生了一些重要变化，粒缺伴发热的病原体尤其是耐药菌相关的流行病学数据也有所更新。依据国内外循证医学证据，中华医学会血液学分会和中国医师协会血液科医师分会组织相关领域专家对2020年版指南进行更新，亦参照GRADE方法进行证据质量评价和推荐意见强度分级。

## Abstract (en)

Febrile neutropenia is associated with a high risk of infection-related mortality if appropriate antimicrobial therapy is not initiated promptly. In recent years, the diagnostic and therapeutic concepts for febrile neutropenia have substantially advanced, along with new epidemiological data on pathogens, particularly drug-resistant bacteria. According to evidence-based medical evidence from both domestic and international sources, the Chinese Society of Hematology, Chinese Medical Association and Chinese Medical Doctor Association, Hematology Branch organized experts in related fields to update the 2020 edition guidelines. The GRADE method was employed to develop revised recommendations for evidence quality assessment and recommendation strength grading.

中性粒细胞缺乏（粒缺）伴发热患者是一组特殊的疾病人群。由于免疫功能低下，炎症相关临床症状和体征常不明显，病原体及感染灶也不明确，发热可能是感染的唯一征象，如未及时给予恰当的抗菌药物治疗，感染相关死亡率高。因此，充分认识粒缺伴发热患者的相关风险、诊断方法及如何合理使用抗菌药物对于降低粒缺伴发热的发生率和死亡风险至关重要。

《中国中性粒细胞缺乏伴发热患者抗菌药物临床应用指南（2020年版）》的发表对于临床诊疗发挥了重要的指导作用。近年来，国际及国内关于粒缺伴发热的诊疗理念发生了一些重要变化，我国在粒缺伴发热的病原体，尤其是耐药菌监测方面也积累了大量临床研究和流行病学数据，新型靶向药物及免疫治疗的应用带来新的危险因素。因此，参考欧洲白血病感染相关指南（ECIL指南）[1]–[4]、美国感染病学会（IDSA）肿瘤合并粒缺患者治疗指南（IDSA指南）[5]、2019年西班牙血液恶性肿瘤患者粒缺伴发热管理共识[6]、《血液肿瘤患者碳青霉烯类耐药肠杆菌科细菌（CRE）感染的诊治与防控中国专家共识（2025年版）》[7]等，结合我国当前细菌流行病学、耐药监测数据及抗菌药物临床应用经验总结，中华医学会血液学分会和中国医师协会血液科医师分会组织血液病学、微生物学、药学、统计学领域专家依据GRADE方法对2020年版指南进行修订。

一、方法学

1. 指南目的：为粒缺伴发热患者的临床规范化诊疗、科学合理使用抗菌药物提供参考。

2. 指南制订工作组：启动时间为2025年4月12日，定稿时间为2026年3月23日。本指南的制订工作组由血液科、呼吸与危重症医学科、药学专家、微生物学专家及循证医学等多学科专家组成。制订工作组成员按照主要职能分为牵头专家、指导专家、执笔专家、共识组、证据评价组和秘书组。所有制订工作组成员均不存在与本指南相关的经济或非经济利益冲突。制订过程或推荐意见形成未受到资助影响。

3. 指南注册：本指南在制订初期撰写了计划书，并在国际实践指南注册与透明化平台（http://www.guidelines-registry.cn）进行了中英文双语注册（注册号：PREPARE-2026CN631）。

4. 指南的使用者与目标人群：本指南的使用者为血液科医师、感染科医师、肿瘤科医师、临床药师、重症医学科医师及其他相关临床医务人员。目标人群为指南涉及的出现粒缺［中性粒细胞绝对计数（ANC）<0.5×10^9^/L］伴发热（体温≥38.3 °C或≥38.0 °C持续1 h以上）的患者，主要为血液系统疾病、恶性肿瘤化疗等导致的粒缺患者。

5. 临床问题调研与遴选：本指南的临床问题遴选分为3个步骤：①首先系统查找和阅读粒缺伴发热患者临床治疗领域已发表的指南、临床试验、临床研究，并结合对部分临床医师的调研和访谈结果，初步拟定了15个临床问题。②通过问卷星对指南专家进行临床问题重要性调研（采用Likert 5分量表：5分：很重要；4分：比较重要；3分：一般重要；2分：不太重要；1分：不重要），同时补充重要但尚未被纳入的临床问题。③召开专家组会议，对临床问题调研结果进行讨论，最终确定本指南所关注的12个临床问题。

6. 证据检索与筛选：按照人群、干预、对照、结局（PICO）原则，证据评价组对需解决的12个临床问题进行解构，分别制订包含主题词和自由词的检索策略并进行系统检索，检索范围包括：①PubMed、Embase、The Cochrane Library、Web of Science等英文数据库；②中国知网、维普、万方等中文数据库；③美国食品药品监督管理局（FDA）、欧洲药品管理局（EMA）和NMPA官网发布的抗菌药物说明书，并收集欧洲白血病感染会议（ECIL）相关指南、美国感染病学会（IDSA）肿瘤合并粒缺患者治疗指南、2019年西班牙血液恶性肿瘤患者粒缺伴发热管理共识及中华医学会等权威学术团体的相关指南与共识、ECIL/IDSA等国际会议。检索时限涵盖建库至2025年7月30日，主要纳入系统评价、荟萃分析、随机对照试验、队列研究、病例对照研究、病例系列、病例报告和指南/共识等。完成文献检索后，针对每个临床问题，均由证据评价组成员按照文献题目、摘要和全文的顺序逐级独立筛选文献，确定纳入符合具体临床问题的文献（发表语言限定为中、英文）。

7. 证据分级：采用GRADE进行证据质量评价和推荐意见强度分级。证据质量分为高（A）、中（B）、低（C）、极低（D）四级，并将推荐意见等级分为强推荐（1）和弱推荐（2）（表1）[8]–[9]。

**表1:** 推荐意见的证据等级和推荐强度

项目	说明
证据质量	
高（A）	非常有把握：观察值接近真实值
中（B）	对观察值有中等把握：观察值有可能接近真实值，但亦有可能差别很大
低（C）	对观察值的把握有限：观察值可能与真实值有较大差别
极低（D）	对观察值几乎无把握：观察值与真实值可能有极大差别
推荐强度	
强（1）	明确显示干预措施利大于弊或弊大于利
弱（2）	利弊不确定或无论证据质量高低均显示利弊相当

8. 推荐意见的形成：指导专家、秘书组及证据评价组基于国内外现有证据情况，同时考虑中国患者的偏好与价值观、干预措施的成本、利弊和可及性等，通过讨论初步形成符合我国临床诊疗实践的推荐意见，于2025年12月2日进行推荐意见的调查（强推荐/弱推荐/不推荐），以问卷形式（**扫描本文首页二维码查阅附表1**）达成对推荐意见的分级，原则为：①若强推荐票数超过50％，则视为达成，推荐强度为“强推荐”；②若弱推荐票数超过50％，则视为达成，推荐强度为“弱推荐”；③若强推荐及弱推荐总票数超过70％，亦视为达成共识，推荐强度则直接定为“弱推荐”。在现有证据的基础上，指导专家、秘书组及证据评价组对专家提出的修改意见进一步讨论、修改和完善。

9. 指南意见的撰写、外审及批准：推荐意见达成共识后，由执笔专家完成指南全文撰写，经所有指南专家组成员同意后，邀请外审小组进行同行评审，并对外审意见进行相应修订与完善，最终提交指导委员会审议通过后确定指南终稿。

10. 指南发布与更新：本指南将通过专业期刊、网络及各类学术会议进行推广与发布，确保临床医师及其他利益相关群体充分了解本指南，促进临床医师和相关从业人员对指南内容的规范理解与合理应用。

二、定义

1. 粒缺：指外周血ANC<0.5×10^9^/L，或预计48 h后ANC<0.5×10^9^/L；严重粒缺指ANC<0.1×10^9^/L。

2. 发热：指单次口腔温度≥38.3 °C（腋温≥38.0 °C），或口腔温度≥38.0 °C（腋温≥37.7 °C）持续超过1 h。粒缺期间应避免测定直肠温度，以防止定植于肠道的微生物侵入。

需要指出的是，发热是粒缺患者应用抗菌药物的指征，由于这群患者临床表现差异较大，临床医师的判断在决定是否需要给患者使用抗菌药物治疗时起关键性作用。即使患者不能满足上述定义，也需要医师仔细甄别是否需要应用抗菌药物治疗，如全身状况不良的患者（尤其是老年患者）应警惕感染时可能无发热或表现为低体温。

三、流行病学

1. 粒缺伴发热的流行病学：超过80％的血液肿瘤和10％～50％的实体肿瘤患者在≥1个疗程化疗后会发生与粒缺有关的发热。近年来，免疫治疗及靶向药物广泛应用所致粒缺伴发热亦不少见[10]。血液肿瘤患者粒缺伴发热常有较高的死亡率，其血流感染（BSI）的相关死亡率为7.1％～42％[11]–[13]。粒缺伴发热患者的临床表现往往不典型，感染部位不明显或难以发现，病原体培养阳性率低。能明确感染部位者占50％左右，最常见的感染部位是肺，其次为上呼吸道、肛周和BSI等[13]。我国粒缺伴发热的病原流行病学资料大多来源于BSI数据，与国外调查结果基本一致。致病菌以革兰氏阴性杆菌为主，占62.7％～76.6％[14]–[19]。常见革兰氏阴性杆菌包括大肠埃希菌、肺炎克雷伯菌、铜绿假单胞菌、嗜麦芽窄食单胞菌和鲍曼不动杆菌；常见革兰氏阳性球菌包括链球菌属、金黄色葡萄球菌、凝固酶阴性葡萄球菌和肠球菌。国内调查显示，血液肿瘤或造血干细胞移植患者合并侵袭性真菌感染（确诊及临床诊断）的发生率为2.1％～7.7％[20]–[22]。粒缺患者病毒感染主要包括社区获得性呼吸道病毒感染和疱疹病毒感染/激活。社区获得性呼吸道病毒包括流感病毒、副流感病毒、新型冠状病毒、呼吸道合胞病毒、腺病毒、鼻病毒、其他冠状病毒等[23]，流行季节普遍易感。疱疹病毒包括巨细胞病毒、EB病毒、水痘-带状疱疹病毒等，粒缺及免疫功能低下患者可发生感染或再激活[10]。病原谱因感染部位和危险因素不同存在差异[11],[14],[24]–[26]；此外，不同地区及医院、科室也存在差异[11],[15],[18]–[19],[27]–[29]。

2. 耐药菌感染的流行病学：粒缺伴发热患者超过半数的耐药菌从BSI中检出，而呼吸道感染的耐药菌检出率较低。近5年血液科BSI患者产超广谱β-内酰胺酶（ESBL）大肠埃希菌（产ESBL-EC）、产ESBL肺炎克雷伯菌（产ESBL-KP）、碳青霉烯类耐药大肠埃希菌（CR-EC）、碳青霉烯类耐药肺炎克雷伯菌（CRKP）、碳青霉烯类耐药铜绿假单胞菌（CRPA）、碳青霉烯类耐药鲍曼不动杆菌（CRAB）发生率分别为46.55％～57.53％、31.57％～47.50％、3.50％～14.00％、8.77％～30.30％、6.10％～16.67％、25.80％～60.00％[15]–[17],[30]–[33]。与欧美国家相比，我国整体人群CRE感染的发生率较高且逐年增加，2025年为25.5％（CHINET中国细菌耐药监测网数据），是粒缺伴发热目前面临的挑战[7],[34]。我国CRE以产KPC酶及NDM酶为主，部分产IMP酶和OXA酶。不同CRE菌株的产酶方式不同，大肠埃希菌以产NDM酶为主，肺炎克雷伯菌以产KPC酶为主，其次为NDM酶[35]–[36]。中国医院侵袭性真菌病监测网（CHIF‑NET）2019–2021年的血液标本药敏监测数据显示，白念珠菌和近平滑念珠菌对唑类药物总体保持敏感，热带念珠菌对伏立康唑的敏感性为49.4％，光滑念珠菌对伏立康唑的敏感性为58.0％[37]。曲霉菌对现有抗真菌药物耐药率呈上升趋势[38]–[39]。

尽管相当一部分粒缺伴发热患者最终无法明确致病病原体，考虑到这类患者病情严重、死亡率较高，尽早开始抗菌药物治疗可显著改善粒缺伴发热患者的预后；同时，运用多种病原学检测方法明确病原体对目标性抗感染治疗至关重要。

四、诊断

1. 病史询问和体格检查：评估患者状态，详细了解既往抗生素应用、耐药和定植情况，寻找感染的高危和隐匿部位（1B）；但相当一部分患者无法明确感染部位。

2. 实验室检查：全血细胞计数、肝肾功能和电解质检查，至少每3天复查1次；降钙素原（PCT）、C反应蛋白等感染相关指标的检查对感染诊断有提示意义（1C）。对于粒缺患者，PCT的诊断意义可能有限[28]。

3. 微生物学检查：至少同时行两套血培养检查，如果存在中心静脉导管（CVC），一套血标本从CVC的管腔采集，另一套从外周静脉采集。无CVC者，应采集不同部位静脉的两套血标本进行培养，采血量为每瓶10 ml。如果经验性抗菌药物治疗后患者仍持续发热，可以每隔2～3 d进行1次重复培养。同时根据临床表现，对可能出现感染的部位进行相应的微生物学检查。除培养外，根据疾病情况，其他微生物学检测方法也应当进行，包括：

（1）临床样本的涂片镜检：采集感染部位组织或组织分泌物，如下呼吸道标本、脑脊液、脓液等进行涂片镜检，对病原学诊断有一定参考价值，可作为初始经验性抗感染治疗的依据（1C）。

（2）血清学检测：急性期血清学IgM抗体阳性对诊断有指导价值，恢复期IgG抗体滴度呈4倍或4倍以上变化或IgM抗体由阴性转为阳性具有回顾性诊断的价值。但粒缺患者由于免疫功能低下，急性期血清学阳性检出率低。血清（1，3）-β-D-葡聚糖试验（G试验）、血清或肺泡灌洗液半乳甘露聚糖抗原试验（GM试验）阳性对侵袭性真菌病有辅助诊断价值（1A）[20],[40]–[44]。近年来开展的真菌血清学5G+联合检测包括G试验、GM试验、隐球菌荚膜多糖抗原（GXM）、曲霉IgG抗体试验、念珠菌IgG抗体试验、念珠菌甘露聚糖抗原检测（Mn试验）和曲霉特异性IgE试验等[45]，可能有助于侵袭性真菌病的精准诊疗，但其在粒缺患者中的应用有待探讨。

（3）聚合酶链反应（PCR）和宏基因组二代测序（mNGS）：PCR检测血液或组织中微生物DNA/RNA含量，对细菌、真菌、病毒及特殊病原体等具有诊断价值（1A）[20],[46]–[48]。mNGS通过检测临床标本中微生物的DNA/RNA结合相对丰度判断致病菌，可提高病原检测的敏感性，缩短检测时间，在罕见病原体感染的诊断中具有优势（1B）[49]–[56]。对于血液病患者，mNGS可获得更全面、相对无偏倚的病原信息，尤其对于传统微生物学检测未覆盖或检测周期较长、阳性率较低的病原可提高检出率。考虑到其目前成本仍较高，应对检测适应证进行规范和限定，其主要应用领域仍然是急、危、重、难的患者感染诊治，避免过度使用[49]。对于有上呼吸道症状（如卡他性鼻炎）和（或）咳嗽患者，应进行呼吸道病毒核酸或抗原检测（包括流感病毒、副流感病毒、新型冠状病毒、腺病毒、呼吸道合胞病毒和人偏肺病毒等检测）（1A）[57]–[58]。

4. 相关感染部位的评估和影像学检查：X线、CT、B超、PET-CT等。PET-CT可用于鉴别诊断、排查感染灶及评估疗效（2C）[59]。

诊断流程见图1。

**图1:**
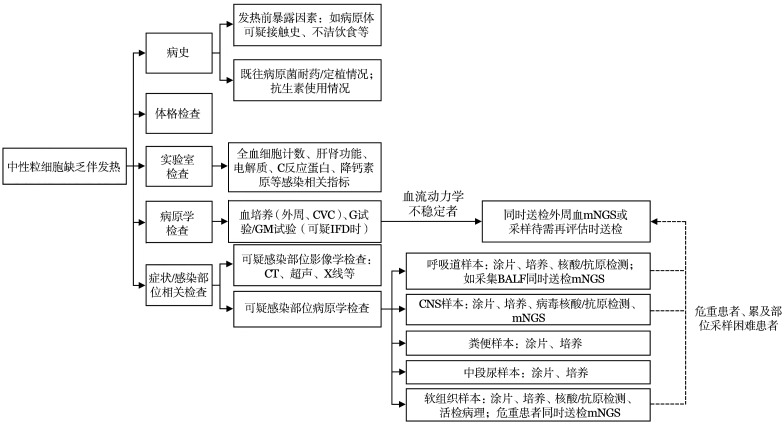
中性粒细胞缺乏伴发热患者诊断流程图 **注** CVC：中心静脉导管；G试验：血清（1，3）-β-D-葡聚糖试验；GM试验：半乳甘露聚糖抗原试验；IFD：侵袭性真菌病；mNGS：宏基因组二代测序；BALF：支气管肺泡灌洗液；CNS：中枢神经系统

五、患者的危险分层和耐药评估

危险分层是粒缺伴发热患者治疗开始前的必要工作，对于后续经验性选择抗菌药物至关重要（表2）（1A）[1],[3],[5]，危险分层包括高危和低危，高危患者必须住院治疗，不符合低危标准的患者均应按照高危患者处理。

**表2:** 中性粒细胞缺乏伴发热患者的危险分层

危险度	定义
高危	符合以下任意一项： 1. 预计严重中性粒细胞缺乏（中性粒细胞绝对计数<0.1×10^9^/L）持续>7 d。 2. 有以下任意一种临床合并症：①血流动力学不稳定；②口腔或胃肠道黏膜炎，吞咽困难；③胃肠道症状：腹痛、恶心、呕吐和腹泻；④新发的神经系统改变或精神症状；⑤血管内导管感染，尤其是导管腔道感染；⑥新发的肺部浸润或低氧血症，或有潜在的慢性肺部疾病。 3. 肝功能不全（转氨酶水平>5倍正常上限）或肾功能不全（肌酐清除率<30 ml/min）。 4. 合并免疫功能缺陷疾病。 5. 接受分子靶向药物或免疫调节药物治疗。
低危	预计中性粒细胞缺乏时间≤7 d，无活动性合并症，肝肾功能正常或损害较轻且稳定

随着抗菌药物耐药问题日趋严重，粒缺伴发热患者在经验性治疗前还应进行耐药危险因素评估（表3）（1A）[1]。

**表3:** 中性粒细胞缺乏伴发热患者耐药细菌感染的危险因素

1. 患者有耐药病原菌定植或感染病史，尤其是：①产超广谱β-内酰胺酶或碳青霉烯酶的肠杆菌；②耐药非发酵菌：铜绿假单胞菌、鲍曼不动杆菌、嗜麦芽窄食单胞菌；③耐甲氧西林金黄色葡萄球菌，尤其是万古霉素最低抑菌浓度≥2 mg/L；④耐万古霉素肠球菌。
2. 接触过广谱抗菌药物，尤其是第三代头孢菌素类、喹诺酮类。
3. 重症疾病：如晚期肿瘤、脓毒血症、肺炎。
4. 院内感染。
5. 长期和（或）反复住院。
6. 留置导管。
7. 老年患者。
8. 重症监护病房患者。

六、初始经验性抗菌药物治疗

在危险分层和耐药危险因素评估后，尽快使用抗菌药物初始经验性治疗，而不必等待微生物学的结果，其原则是覆盖可迅速引起严重并发症或威胁生命的最常见和毒力较强的病原体，同时必须考虑本区域、本院及本科室感染的流行病学覆盖耐药菌，直至获得准确的病原学结果。

选择恰当的经验性抗菌药物治疗具有重要临床意义。接受不恰当的初始经验性抗菌药物治疗［IIAT：抗菌药物对致病病原体的体外药敏试验为耐药和（或）中介］可导致感染相关病死率升高[60]–[61]。因此，制定合理的经验性抗菌药物治疗方案至关重要。制定经验性抗菌药物治疗方案需要综合评估患者、病原、抗菌药物等多方面因素，选择具有杀菌活性、抗假单胞菌活性和安全性良好的广谱抗菌药物，并需注意与治疗原发疾病的药物（化疗药物、免疫抑制剂等）是否存在不良反应的叠加。

对于低危患者，初始治疗可以在门诊或住院接受口服或静脉注射经验性抗菌药物治疗[1]。对接受门诊治疗的患者需要保证密切的临床观察和恰当的医疗处理，如病情加重须尽快住院治疗。高危患者必须立即住院治疗，根据危险分层、耐药危险因素、当地病原体和耐药流行病学数据及临床表现复杂性对患者进行个体化评估[1]。抗生素升阶梯和降阶梯策略的适应证及经验性抗菌药物选择的建议见表4[2]。

**表4:** 中性粒细胞缺乏（粒缺）伴发热患者升阶梯和降阶梯策略的适应证和经验性抗菌药物选择的建议

治疗策略	适应证	初始抗菌药物选择
升阶梯策略	1. 无复杂临床表现^a^； 2. 不确定有无耐药菌定植； 3. 此前无耐药菌感染； 4. 本中心粒缺伴发热由耐药菌感染导致罕见。	1. 抗假单胞菌头孢菌素（如头孢吡肟、头孢他啶）； 2. β-内酰胺酶抑制剂复合制剂（如头孢哌酮/舒巴坦、哌拉西林/他唑巴坦）； 3. 哌拉西林+阿米卡星。
降阶梯策略	1. 复杂临床表现^a^； 2. 存在耐药菌定植； 3. 发生过耐药菌感染； 4. 本中心粒缺伴发热常见由耐药菌感染导致。	1. 碳青霉烯类单药； 2. 抗假单胞菌β-内酰胺类联合氨基糖苷类或喹诺酮类（重症患者选择β-内酰胺类中的碳青霉烯类）； 3. 早期覆盖革兰氏阳性耐药菌（如果存在革兰氏阳性菌感染风险）：糖肽类、利奈唑胺或新型抗生素。

**注** CRO：碳青霉烯类耐药革兰阴性菌；^a^复杂临床表现包括：血流动力学不稳定、局灶性感染（如肺炎、肠炎、中心静脉导管相关感染）、长期和严重营养不良、并发症（出血、脱水、器官衰竭、慢性病）、高龄（60岁以上）

高危患者静脉应用的抗菌药物必须是能覆盖铜绿假单胞菌和其他严重革兰氏阴性杆菌的广谱抗菌药物（1A）。鉴于目前国内流行病学数据，尤其是耐药菌比例和耐药谱的变化，经验性用药时，还应参照本地区、本医院和本科室最新的耐药菌流行病学数据、感染部位、药物在目标人群中的药代动力学（PK）/药效学（PD）等，尽可能做到准确经验用药。对于既往发生过广泛耐药（XDR）细菌定植或感染的患者，初始经验用药更应慎重。对于有产ESBL菌定植或感染病史及产ESBL菌感染高危患者，选择碳青霉烯类单药或β-内酰胺酶抑制剂复合制剂或β-内酰胺类联合氟喹诺酮类或氨基糖苷类抗菌药物治疗[62]–[71]（1A）。对于存在碳青霉烯类耐药革兰阴性菌（CRO）定植或既往CRO感染的患者，如粒缺伴发热时合并下列情况之一：表现为重症感染（血流动力学不稳定、脓毒症、感染性休克或肺炎）、肠道定植者发生严重黏膜炎或肠梗阻或肛周脓肿、采用数字PCR或mNGS检测血液发现与定植菌相同菌种，宜尽快采用相应经验性治疗[4],[7],[72]–[74]，药物选择参考目标治疗（**扫描本文首页二维码查阅附表2**）。

对于以下特定情形，初始经验性用药需要同时覆盖严重的革兰氏阴性杆菌和革兰氏阳性球菌：

①血液动力学不稳定或有其他严重BSI证据（1B）；

②X线影像学确诊的肺炎（1B）；

③在最终鉴定结果及药敏试验结果报告前，血培养为革兰氏阳性球菌（1A）；

④临床疑有严重导管相关感染（1B）；

⑤任一部位的皮肤或软组织感染（1B）；

⑥耐甲氧西林金黄色葡萄球菌、耐万古霉素肠球菌或耐青霉素肺炎链球菌定植（1B）。

如患者在接受初始抗细菌治疗时即发生血流动力学不稳定，可考虑经验性抗真菌治疗（2C）。在流感流行季节，对可疑流感病毒感染或有相关暴露史患者进行快速核酸检测或直接抗原检测，阳性者予抗病毒治疗（1A）[23],[75]–[76]。

七、抗菌药物的调整

在接受经验性抗菌药物治疗后，应根据危险分层、确诊的病原体和患者对初始治疗的反应等综合判断，决定后续如何调整抗菌治疗。临床上，在初始经验性抗菌药物应用的基础上，如果出现病情加重，如血流动力学不稳定，宜及时调整抗菌药物。对于病原体明确的患者，可根据所识别细菌和药敏结果采用窄谱抗生素治疗，检出细菌如属于耐药菌，应根据病原体及其药敏结果（最低抑菌浓度值）选择针对性抗菌药物，有条件的医院可行耐药基因检测。一般推荐联合抗菌药物治疗耐药菌感染，具体药物选择详见附表2（**扫描本文首页二维码查阅**）。对于接受抗感染治疗72～96 h后未能明确病原体的患者，抗生素的调整流程见图2。在抗细菌药物治疗无效时，需考虑真菌、病毒和其他病原体（包括非典型病原体）感染的可能，参照相关指南和共识尽早开始抗真菌和抗其他病原体的治疗[20],[23],[77]–[78]。

**图2:**
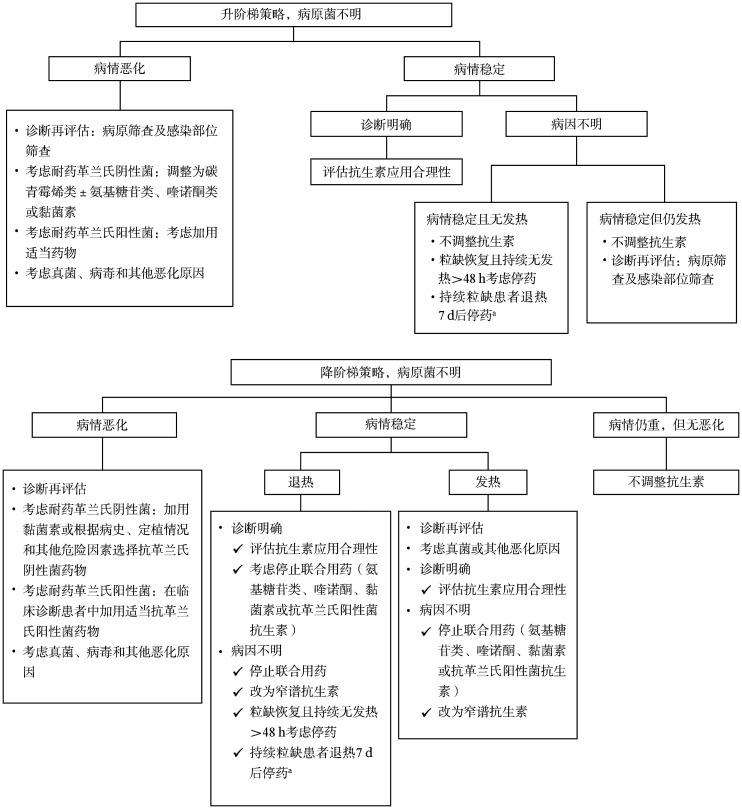
经验性抗菌药物治疗后中性粒细胞缺乏（粒缺）伴发热患者治疗调整流程 **注** ^a^有研究显示，若患者无发热且血流动力学稳定，感染的症状和体征消失，但中性粒细胞绝对计数仍<0.5×10^9^/L，抗生素经验治疗72 h后可考虑停用，但宜严密观察24～48 h，如再出现发热，应在完善临床及病原学评估后立即重新开始抗菌药物治疗

当粒缺伴发热患者接受初始经验性抗细菌药物治疗后无效，或治疗有效后再次发热，需积极完善影像学诊断（如胸部CT）和实验室诊断［包括血培养、血GM试验、PCR和（或）mNGS等］，必要时行支气管镜检查评估侵袭性真菌病（IFD）风险。IFD高危患者以经验治疗为主，即以持续粒缺伴发热且广谱抗菌药物治疗4～7 d无效作为启动治疗的主要标准；IFD低危患者则推荐在出现临床影像学异常（如胸部CT出现曲霉菌感染相关影像学改变）或微生物学标志（如GM/G试验阳性、非无菌部位或非无菌操作所获得的标本真菌培养或镜检阳性）等IFD诊断依据时进行，即诊断驱动治疗。未接受广谱抗真菌药物预防患者的经验治疗药物一般选择覆盖念珠菌或曲霉菌的广谱抗真菌药物，包括伏立康唑、棘白菌素类（卡泊芬净）、艾沙康唑和两性霉素B制剂等药物。接受广谱抗真菌药物（如泊沙康唑/伏立康唑）预防患者，建议进行血药浓度监测提升和（或）维持有效预防治疗浓度。考虑IFD突破患者药物治疗选择仍不明确，根据当地流行病学排除交叉耐药可换用其他三唑类或更换其他类型抗真菌药物如棘白菌素类（卡泊芬净）或两性霉素B脂质体（2C）[79]–[80]。明确IFD患者予目标治疗，治疗方案依据真菌种类和抗真菌药物的抗菌谱进行选择。

粒缺伴发热患者接受初始经验性抗细菌药物治疗后无效，如考虑可疑呼吸道病毒感染或疱疹病毒激活，需完善相应的核酸或抗原检测，必要时完善肺部影像学检查，根据结果予抗病毒药物等治疗（1C）[57],[77],[81]。

八、抗菌药物治疗的疗程

对于粒缺伴发热患者的抗生素使用时长，目前尚无统一的标准。对于不明原因发热的粒缺患者，抗菌药物经验性治疗后如ANC≥0.5×10^9^/L，稳定退热48 h后可考虑停用抗菌药物；如ANC持续<0.5×10^9^/L，抗菌药物可用至退热7 d后停药。一些研究显示，如患者无发热且血流动力学稳定，感染的症状和体征消失，但ANC仍<0.5×l0^9^/L，抗生素经验治疗72 h后可考虑停用，但宜严密观察24～48 h，如果再次出现发热，应在完善临床及病原学评估后立即重新开始抗菌药物治疗[82]–[83]（1B）。微生物证实及临床证实的感染治疗疗程取决于特定的微生物和感染部位[4],[59],[84]–[87]，详见表5。

**表5:** 中性粒细胞缺乏伴发热患者微生物证实及临床证实的感染治疗疗程

感染类型	疗程
细菌性肺炎	7～14 d
细菌性鼻窦炎	7～14 d
皮肤软组织感染	7～14 d
腹部复杂感染	感染证据完全消失
存在深部组织感染、心内膜炎、化脓性血栓性静脉炎或接受适当抗菌药物治疗拔除导管后仍有持续性（>72 h）血流感染	>4周或至病灶愈合、症状消失
革兰氏阴性杆菌血症	10～14 d
革兰氏阳性球菌血症	7～14 d（复杂感染及特殊病原菌需治疗较长时间）
耐甲氧西林金黄色葡萄球菌血流感染	糖肽类药物、达托霉素等治疗至少14 d，合并迁徙性病灶者适当延长
耐甲氧西林凝固酶阴性的葡萄球菌或肠球菌引起的血流感染	体温正常后持续治疗5～7 d
导管相关性血流感染	建议拔除导管，未拔除导管者适当延长疗程

九、抗菌药物预防给药的指征

对于高危粒缺患者，可以应用喹诺酮类药物预防细菌感染（2C），但喹诺酮类药物预防仅可降低BSI发生率，对总体死亡率无影响[88]–[91]，预防用药宜充分考虑本地区细菌耐药的流行病学特点及药物不良反应等。最佳的开始给药时间和给药持续时间尚无定论，推荐自粒缺开始应用至ANC>0.5×10^9^/L或出现明显的血细胞恢复的证据（2C）[92]。需要注意的是，长期使用喹诺酮类药物预防可能导致革兰氏阳性球菌感染，并可能导致多药耐药菌株的定植或感染增加及氟喹诺酮耐药菌血症菌株增加。对于低危及多药耐药菌定植的患者不建议预防性应用抗菌药物（1B）[7],[13],[93]。

对于具有IFD高危因素或既往诊断IFD的患者，建议应用抗真菌预防（1A），疗程主要取决于患者IFD高危因素的改善情况[20]。

此次更新主要关注粒缺伴发热病原体流行病学变化、分子学等诊断方法和新型抗生素的应用，同时强调需要结合患者状态及本地病原体流行情况个体化选择适当的初始治疗。及时合理地调整抗生素及控制疗程对避免耐药菌感染率进一步攀升至关重要。然而，目前对于这类患者治疗的研究仍有局限性，一定程度上缺乏高质量等级证据，有待更多前瞻性临床研究探讨。

## Supplementary Material


