# Supplementary figures and images for: Chinese guidelines for the clinical application of antibacterial drugs for febrile neutropenia（2026）
Source: Zhonghua Xue Ye Xue Za Zhi. 2026 Apr;47(4):305–18. [Article in Chinese] doi: 10.3760/cma.j.cn121090-20260120-00041 (PMC13195557; doi:10.3760/cma.j.cn121090-20260120-00041)

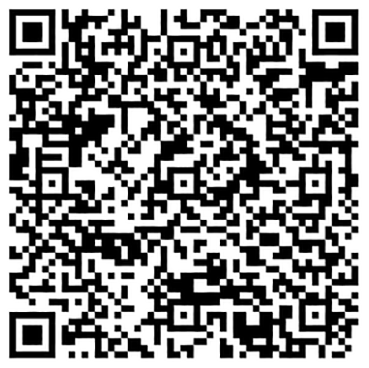

Supplement: Supplementary file 1 [file cjh-47-04-305-g003.tif]
